# An Update on Obstructive Sleep Apnea Syndrome—A Literature Review

**DOI:** 10.3390/medicina59081459

**Published:** 2023-08-13

**Authors:** Alexandra Lorina Platon, Carmen Gabriela Stelea, Otilia Boișteanu, Emilia Patrascanu, Irina Nicoleta Zetu, Sorana Nicoleta Roșu, Valentina Trifan, Dragoș Octavian Palade

**Affiliations:** 1 Department of Orthodontics and Dentofacial Orthopaedics, Grigore T. Popa University of Medicine and Pharmacy, 16 Universitatii Str., 700115 Iasi, Romania; alexandra.lorina.platon@umfiasi.ro (A.L.P.); nicoleta.zetu@gmail.com (I.N.Z.); 2 Department of Oral Surgery, Grigore T. Popa University of Medicine and Pharmacy, 16 Universitatii Str., 700115 Iasi, Romania; 3 Department of Anesthesia and Intensive Care, Grigore T. Popa University of Medicine and Pharmacy, 16 Universitatii Str., 700115 Iasi, Romania; patrascanu.emilia@umfiasi.ro; 4 Department of Community and Oral Health, Grigore T. Popa University of Medicine and Pharmacy, 16 Universitatii Str., 700115 Iasi, Romania; rosu.sorana@umfiasi.ro; 5 Department of Orthodontics and Dentofacial Orthopaedics, Nicolae Testemițanu State University of Medicine and Pharmacy, Stefan cel Mare si Sfant Boulevard 165, 2004 Chișinău, Moldova; valentina.trifan@usmf.md; 6 ENT, 2nd Surgery Department, Grigore T. Popa University of Medicine and Pharmacy, 16 Universitatii Str., 700115 Iasi, Romania; octavian.palade@umfiasi.ro

**Keywords:** obstructive sleep apnea, sleep apnea syndrome, mandibular advancement devices, myofunctional therapy

## Abstract

Obstructive sleep apnea syndrome (OSAS) is the most common breathing-related sleep disorder. It is characterized by recurrent episodes of partial or complete airway obstruction during sleep, resulting in a reduction in or the total cessation of airflow, despite ongoing respiratory efforts, leading to oxygen desaturation and arousal. The purpose of this literature review is to evaluate the most common characteristics of this pathology, as well as to investigate the most effective treatment options, providing an update on the management of OSA patients.

## 1. Introduction

Sleep disorders encompass a wide spectrum of conditions that need to be identified, diagnosed, and treated as early as possible because they have a great impact on and serious consequences for general health. The most common breathing-related sleep disorder is obstructive sleep apnea (OSA).

OSA represents a major health problem in the most developed countries, with approximately 1 billion of the world’s population, aged between 30 and 69 years, suffering from this condition [1]. This disorder was first described in 1965 [2].

According to the American Academy of Sleep Medicine, it is characterized by recurrent episodes of partial or complete airway obstruction during sleep, resulting in a reduction (hypopnea) in or in the total cessation (apnea) of airflow, despite ongoing respiratory efforts, leading to oxygen desaturation and arousal. The final effect is sleep fragmentation, intermittent hypoxia, and hypercapnia, leading to increased sympathetic nervous system activity.

The absence of deep sleep leads to daytime sleepiness, cognitive impairment, and lower quality of life [3]. Also, obstructive sleep apnea has been associated with various health-related consequences such as myocardial infarction, heart failure, hypertension, global cardiovascular morbidity, and diabetes mellitus.

There are bidirectional relationships between endocrine disorders and OSA. Several endocrine disorders are risk factors for OSA (obesity, acromegaly, Cushing syndrome, type 1 and type 2 diabetes, and hypothyroidism), and OSA can also induce endocrine disorders, particularly glucose metabolism abnormalities.

Recently, numerous studies have demonstrated that surgical patients with OSA are at an increased risk of having perioperative complications, including hypoxemia, pneumonia, difficult intubation, pulmonary embolism, cardiac arrhythmias, and unanticipated ICU admission.

The majority of patients with OSA are undiagnosed.

The aim of this literature review is to provide a concise overview of the current state of OSA research and its importance in the medical field, summarizing the most relevant and up-to-date information regarding its symptomatology, and prevalence, as well as the most effective diagnostic and treatment tools.

## 2. Material and Methods

This literature review about OSA was realized using the PUBMED database. The words used to research articles were obstructive sleep apnea, sleep apnea syndrome, mandibular advancement devices, and myofunctional therapy. We identified approximately 24,500 articles that were published in the last 5 years, and we evaluated those which contained useful information about epidemiology, pathogenesis, diagnosis, and treatment. We also considered other articles published or reviews with no impact factor.

## 3. Discussion. Review of Findings

### 3.1. Epidemiology

It is estimated that 2–4% of men and 1–2% of women from the general population are affected by OSA [4], but the prevalence is higher in some categories, such as obese patients and ones who have suffered a stroke [5]. In the United States of America, the estimated prevalence of OSA in middle-aged adults is 10% for mild OSA, 3.8% for moderate OSA, and 6.5% for severe OSA. However, it is estimated that nearly 80% to 90% of patients from the USA with OSA remain undiagnosed because of low awareness of this condition among the general public and health professionals [6].

### 3.2. Pathogenesis

IN the upper airway, the region where most obstructive phenomena linked to OSA are found is represented by the pharynx, especially the oropharynx and hypopharynx. The main cause of OSA is a reduction in the expansion forces of the pharyngeal dilatator muscles, in situations such as genioglossal muscle dysfunction, and discoordination between the inspiratory activity of the muscle and respiratory effort, which play an important role in the evolution of this condition [7].

### 3.3. Risk Factors

**Figure 1:**
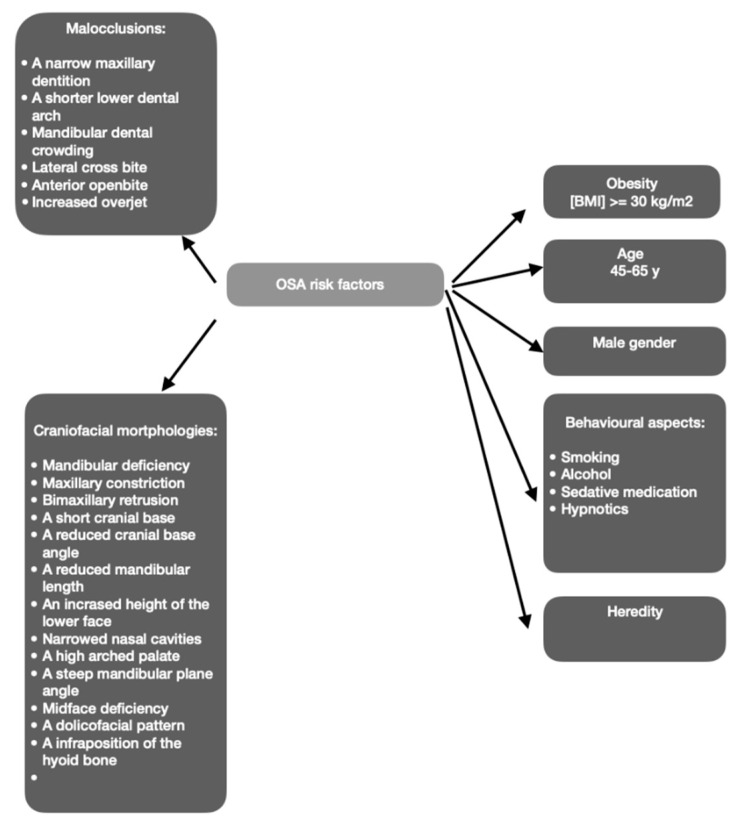
Risk factors for OSA.

There are some conditions that may represent risk factors (Figure 1) for the development of this pathology in the adult population, such as obesity (body mass index (BMI) ≥ 30 kg/m^2^), the menopause, male gender, and increasing age [5]. Snoring and apneas are signs of increased resistance of the upper airway. This usually happens because of some anatomical factors that narrow the upper airway, such as being overweight with increased fat surrounding the pharynx, macroglossia, a retropositioned tongue due to a mandibular retrognathia [3], excessive or elongated soft palatal tissues, tonsillar hypertrophy, and a redundant pharyngeal mucosa [7]. Other risk factors that are associated with OSA include endocrine disorders (an underactive thyroid gland, higher levels of testosterone), personal habits (alcohol, sedatives, and smoking) [8], and heredity [3].

#### 3.3.1. Obesity

Obesity (body mass index (BMI) ≥ 30 kg/m^2^) [5] with central body fat distribution is one of the main components contributing to OSA [9]. Obesity is associated with poor quantity and quality of sleep [10]. Although obesity is regarded as a principal risk factor for OSA [11], it has been shown that the neck perimeter is more highly correlated to the severity of this syndrome than BMI, even though these parameters are usually directly proportioned [7]. But, despite the association between OSA and obesity, it is important to underline the fact that many slender people are diagnosed with OSA [3].

#### 3.3.2. Age and Gender

The prevalence of OSA continuously increases with age for both men and women, with later onset in women [12]. The literature suggests that people aged between 45 and 65 years have a higher risk of developing OSA. In what concerns gender, the prevalence of OSA in men is two or three times higher than in women [13]. Even though overweight middle-aged men have the highest prevalence of OSA, there is an increasing number of women and children who are diagnosed with this condition [14].

#### 3.3.3. Craniofacial Morphologies

Mandibular deficiency, bimaxillary retrusion, a short cranial base, a reduced cranial base angle, a reduced mandibular length, an increased lower anterior facial height, narrowed nasal cavities, maxillary constriction [15], a dolichofacial pattern, a high-arched palate, a steep mandibular plane angle, midface deficiency, and an infraposition of the hyoid bone [5] may predispose to OSA.

#### 3.3.4. Malocclusions Related to OSA

Although orthodontists play an important role in the management of OSA patients, the influence of malocclusions in the development of this pathology is incompletely elucidated. Some studies suggest that a narrow maxillary dentition, a reduced overbite, a shorter lower dental arch, and crowding in the mandibular arch are predisposing factors for OSA. Also, class II malocclusions, a lateral crossbite, an increased overjet, and an anterior open bite have been reported as dental features that are linked to this condition [15].

#### 3.3.5. Smoking, Alcohol, Sedatives, and Hypnotics

Smoking is a risk factor related to OSA in a dose–response relationship. Several surveys have found that smokers and passive smokers have a higher risk of developing snoring compared to non-smokers [3]. Alcohol consumption is common in patients diagnosed with OSA. It is suggested that this habit may cause or exacerbate OSA, especially if the alcohol is consumed shortly before bedtime [16]. Sedative medication and hypnotics are contraindicated for OSA patients due to the concerns of pharyngeal muscle relaxation and delayed arousal worsening hypoxemia, but the information existent in the literature lacks human data [17], so further investigations need to be carried out.

#### 3.3.6. Heredity

Taking into consideration that some studies have reported a significantly higher prevalence of sleep-disordered breathing in relatives of OSA patients than controls, it has been suggested that heredity plays a certain role in the development of this condition [3].

### 3.4. Main Features of OSA

#### 3.4.1. Nocturnal Symptomatology

**Figure 2:**
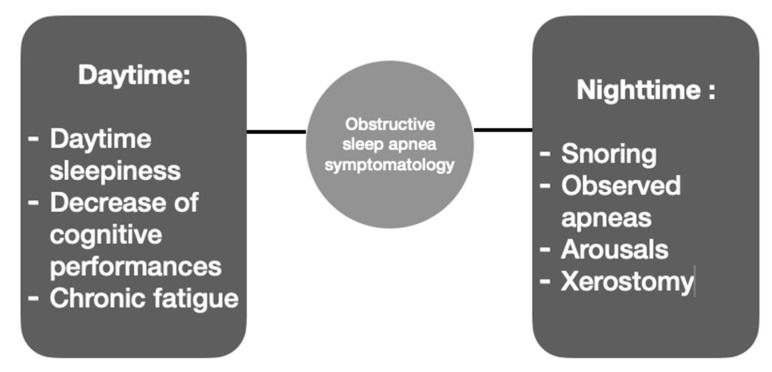
Obstructive sleep apnea symptoms.

Regarding the characteristic symptomatology (Figure 2), a distinction has to be made between day and night.

The most important nocturnal symptoms are snoring, apneas observed by the entourage, and arousal or awakening accompanied by a brief and intense dyspnea sensation [7]. Other nocturnal symptoms related to OSA are choking, diaphoresis, nocturne restless sleep, somniloquy [7], xerostomia, salivation [2], and bruxism [5].

##### Snoring

Snoring is the most important and common symptom of OSA. It is an expression of pharyngeal narrowing [2]. Frequently, it is observed and reported by a bed partner or a member of the entourage [5]. It is estimated that up to 95% of OSA patients have habitual snoring [7]. Patients with OSA usually have a long history of snoring, with increasing intensity, which becomes irregular over time, often in association with increased body weight, alcohol, and sedative consumption or the menopause in women [7]. Various studies have reported that compared to spouses from the general population, spouses of patients with heavy snoring and OSA are more likely to report sleep disturbances [18].

##### Observed Apneas

Observed apneas are usually linked to an increased apnea/hypopnea index (AHI) compared to snoring or excessive daytime sleepiness [7]. If repetitive apnea occurs in a patient many times a night for several years in a row, there will be important changes in the nervous system, myocardial and cerebral circulation, and in pulmonary and systemic circulation [2].

##### Arousals

Arousals and awakenings are conscious phenomena that are accompanied by short and intense dyspnea sensations [7]. Cortical arousals and awakenings are caused by increased work breathing. Repeated arousals increase sympathetic neural activity [5] and produce sympathetic discharges [7], leading to a higher heart rate and blood pressure and a predisposition to cardiac arrhythmia [5].

##### Xerostomia

The prevalence of xerostomia is higher in patients with OSA, especially upon waking up. This symptom is more frequent in persons with moderate OSA compared to those with mild manifestations [19].

#### 3.4.2. Daytime Symptomatology

In what concerns daytime symptomatology, patients with OSA often describe excessive daytime sleepiness, fatigue, morning headaches, a decrease in cognitive performance (lack of attention and difficulties in concentrating), mood disturbances, and difficulty controlling other medical comorbidities such as obesity, type 2 diabetes, and hypertension [5]. Depression, apathy, memory loss, decreased libido [7], and gastroesophageal reflux [2] are also related to OSA.

##### Daytime Sleepiness

OSA can lead to excessive daytime sleepiness and cognitive disorders [20]. Daytime sleepiness is a common complaint in patients with sleep apnea [21] and is the most important diurnal symptom of OSAS [7]. Excessive daytime sleepiness is a consequence of poor quality and quantity of sleep due to the micro-awakenings caused by upper airway obstruction [13]. Excessive daytime sleepiness leads to a higher risk of cognitive impairment, mood disturbances [22], motor vehicle accidents [5], and weight gain [10] and, overall, diminishes quality of life [5].

##### Decrease in Cognitive Performance

If OSA remains undiagnosed, it can lead to decreased cognitive performance with consequent worsening of life quality. This condition is associated with a higher risk of road accidents and home- and work-related injuries [13].

##### Chronic Fatigue

Fatigue is an important aspect in the management of OSA and it is linked to increased, severe dysfunction compared to daytime sleepiness [12].

### 3.5. Diagnosis

The management of an OSA patient must be carried out by a multidisciplinary team with specialists from different healthcare fields: pneumology, cardiology, neurology, ENT, psychiatry, orthodontics and dentofacial orthopedics, and oral maxillofacial surgery [11].

A correct diagnostic algorithm for a patient suspected of OSA includes a complete anamnestic exam, a clinical examination of the patient, the use of sleep questionnaires, cardio-respiratory polygraphy, or, if necessary, overnight polysomnography, auto-CPAP titration if the OSA diagnosis is confirmed, with periodic monitoring of the patient in the outpatient department [11]. Radiographic examinations are also very important for the diagnosis of OSA.

#### 3.5.1. Overnight Polysomnography. Cardio-Respiratory Polygraphy

Overnight polysomnography (PSG) is the gold standard for a proper diagnosis of OSA. This test combines the results of the electroencephalogram, electrocardiogram, electrooculogram, and electromyography together with the patient’s respiratory rate, tidal volume, and inspiratory and expiratory volumes in order to calculate the apnea–hypopnea index (AHI) of the patient [14]. Another diagnostic tool for OSA is represented by home sleep apnea testing (HSAT) [14]. The cardio-respiratory polygraphy is a type of HSAT. It is an effective exam, with a lower price, which can be used at home to detect patients with OSA [23]. This tool is as safe as the PSG in what concerns the determination of the presence and the characteristics of the apneas or hypopneas. The major disadvantage of this method is that it does not record the electroencephalogram, so it is impossible to be certain whether the patient is actually sleeping, so it is mandatory to ask the patient the next day about their sleep quality from the previous night [11]. Given the fact that relying on the patient’s opinion regarding their sleep quality might be a major drawback when analyzing the results of the sleep test, the use of the PSG with a recording of respiratory variables such as AHI/RDI and levels of oxygen saturation [21] remains the gold standard for OSA diagnosis.

According to the International Classification of Sleep Disorders, OSA can be diagnosed using one of the following sets of criteria. The first diagnostic set encompasses the presence of at least one of the following criteria: (1) sleepiness, non-restorative sleep, chronic fatigue or insomnia; (2) waking up with a dyspnea sensation, gasping, or choking; (3) the bed partner or a member of the entourage reports habitual snoring, apneas, or both during the patient’s sleep; (4) the patient suffers from hypertension, coronary artery disease, stroke, congestive heart failure, atrial fibrillation, type 2 diabetes mellitus, a mood disorder, or cognitive dysfunction, and the polysomnographic or the HSAT exam indicates a minimum of five predominantly obstructive events (obstructive or mixed apneas, hypopnea, and respiratory effort-related arousals) per hour of sleep during the PSG or per hour of monitoring during the HSAT. The second set of diagnostic criteria assumes that OSA can be diagnosed if the PSG or the HSAT exam shows at least 15 predominantly obstructive events per hour of sleep during the PSG or per hour of monitoring during the HSAT [5].

#### 3.5.2. Questionnaires

Screening tools such as the Epworth Sleepiness Scale, the Berlin Questionnaire, and the STOP-Bang Questionnaire have been widely used in order to detect OSA.

The Epworth Sleepiness Scale contains a set of simple questions regarding the drowsiness onset to which the patient responds by grading from 0 to 3 (never, rare, moderate, frequent). The values obtained can vary from 0 to 24 points. It is recommended that the patient seeks advice from a specialist if they obtain a score above 10 points [11].

The assessment of the OSA risk factors is achieved by completing the Berlin Questionnaire, which rates the patient’s life quality. This questionnaire contains 10 questions divided into 3 categories: snoring, daily drowsiness, and the presence of obesity or hypertension [24].

The STOP Questionnaire encompasses four “yes/no” questions based on its acronym: S—snoring, T—tiredness, O—observed pauses in breathing, and P—high blood pressure. A score of ≥2 is characteristic of an intermediate risk of OSA [24].

Another validated tool for OSA risk assessment in adults is the STOP-Bang Questionnaire, which incorporates the STOP plus the following sections: B—BMI > 35 kg/m^2^, A—age > 50 years, N—neck circumference ≥ 16 inches in women or ≥17 inches in men, and G—male gender. Low risk of OSA is considered if the questionnaire has a maximum of two “yes” answers, an intermediate risk is when the questionnaire contains three or four “yes” answers, and a high risk is characteristic of five or more “yes” answers. It is considered that the patient has a high risk of developing OSA if there are two “yes” answers from the STOP section together with either high BMI, great neck circumference, or male gender. The STOP-Bang Questionnaire can be completed in a few minutes by the patient [5].

Some studies suggest that compared to the Epworth Sleepiness Scale, the Berlin Questionnaire, and the STOP Questionnaire, the STOP-Bang Questionnaire is more accurate and has a higher sensitivity and diagnostic odds ratio for detecting OSA [24].

#### 3.5.3. Imaging Examinations

Imaging examinations can provide important information when evaluating OSA, but these tools have some disadvantages due to the fact that conventional cephalometric images are dimensionally limited [5].

Lateral cephalometrics are an auxiliary diagnostic tool with an important contribution regarding skeletal and soft tissue morphology. Lateral teleradiographies bring important information about the relationship between OSA and craniofacial and pharyngeal anatomy and have helped to establish some clinical recommendations on the management of these patients [25]. But this auxiliary diagnostic method does not present mediolateral information in the oropharyngeal airway, and it might provide confusing information on the volume and minimal cross-sectional area [5]. Given the limitations of conventional lateral cephalometrics, it is recommended for cone-beam computed tomography (CBCT) or magnetic resonance imaging (MRI) to be used when assessing the presence and severity of OSA.

Cone-beam computed tomography (CBCT) represents a widely used oral and maxillofacial diagnostic tool that provides a three-dimensional view of the hard- and soft-tissue structures of the head and neck [26], which is capable of acquiring quality images that are useful in the analysis and diagnosis of the upper airways. Evaluating the upper airways’ volume in OSA patients is mandatory because it has been demonstrated that these patients have a reduced upper airway volume. The CBCT exam has its maximum utility in assessing the severity of the upper airways’ constriction and in the identification of the OSA etiological factors [27], but it can also be used for monitoring or treating the patient [5].

Another useful imaging examination is represented by magnetic resonance imaging (MRI). It evaluates the soft tissues surrounding the upper airways and it helps in investigating the OSA pathogenesis. MRI exams are also useful when assessing the changes that occur in the upper airways after mandibular advancement device treatment [28].

Current clinical data highlight the fact that three-dimensional imaging of the airway should not be used in order to diagnose obstructive sleep apnea because, at the moment, it does not provide a proper risk assessment technique or screening method. This imaging technique should be used only for monitoring or treatment considerations. If these records are included in the orthodontic diagnosis and treatment plan, the specialist must analyze the upper airways and the surrounding structures [5].

#### 3.5.4. Severity of OSA

There are a few different terms that are used to classify the severity of OSA. When performing the PSG, the severity of OSA is determined by the apnea–hypopnea index (AHI) or the respiratory disturbance index (RDI) [29]. When an HSAT is performed, the severity of OSA will be calculated by the respiratory event index (REI) [29].

The apnea–hypopnea index (AHI) indicates the number of apneas and hypopneas per hour of sleep. The respiratory disturbance index (RDI) calculates the number of apneas, hypopneas, and respiratory-effort-related arousals per hour of sleep; thus, it can be used to indicate the frequency of respiratory events in the total recording time rather than the total sleep time when using the PSG exam. Therefore, an OSA patient can have an RDI higher than the AHI. These differences are important to be understood by clinicians and researchers because some publications refer to AHI while others refer to RDI. The severity of OSA is classified using the AHI or RDI per hour of sleep. Mild OSA is characterized by an AHI or RDI ≥5 and <15, the moderate form is given by an AHI or RDI with values ≥15 but <30, while an AHI or RDI >30 characterizes the severe form of OSA. A patient with normal sleep has an AHI or RDI with values of five or fewer events per hour of sleep [5]. An important thing to mention is that when using cardio-respiratory polygraphy instead of overnight polysomnography, the AHI score might be underestimated due to missing possible hypopneas caused by arousals [20].

The respiratory event index (REI) calculates the frequency of respiratory events based on the total recording time and not on the total sleep time [5]. Compared to PSG, the HSAT does not measure the total sleep time as determined by EEG, so it commonly underestimates the frequency of obstructive events per hour [5].

### 3.6. The Impact of OSA on Life Quality

Untreated OSA can lead to important consequences with a great impact on a patient’s life quality. Excessive daytime sleepiness is a risk factor for motor vehicle accidents and home- or at-work accidents. Furthermore, OSA is linked to a higher risk of insulin resistance, hypertension, coronary artery disease, congestive heart failure, myocardial infarction, stroke, and cardiac arrhythmia [5].

### 3.7. Treatment Options

#### 3.7.1. Continuous Positive Airway Pressure (CPAP) Therapy

CPAP therapy is currently considered the gold standard in OSA treatment in adults. This device works as a form of mechanical ventilation [20] that delivers the CPAP through a mask interface and acts as a pneumatic splint that helps maintain the upper airway latency [5]. The positive airway pressure is exerted on the upper airways during inspiration and expiration, reducing the risk of collapse of the upper airways and, thus, lowering the number of apneas and hypopneas. [20] CPAP therapy can decrease the characteristic symptomatology of OSA, especially in patients with severe OSA (AHI ≥ 30) [5].

#### 3.7.2. Orthodontic Management in Adult OSA

One of the most current trends is involving an orthodontics specialist in the complex multi-disciplinary management of OSA patients. Given their experience in craniofacial growth and development, orthodontists can collaborate with general practitioners to ensure adequate treatment for patients with this condition. Thus, orthodontists can contribute to the screening of these patients in order to identify the craniofacial changes related to OSA, and they can assist general practitioners in the treatment of OSA.

Mandibular advancement devices (MADs): MADs are an innovative treatment method for snoring and mild and moderate OSA. The role of these intraoral devices is to improve patients’ sleep quality by reducing or even eliminating snoring and respiratory pauses during sleep. MADs lead the mandible to the anterior and inferior, generating anatomical changes in the upper airways, thus increasing the pharyngeal area. This movement stabilizes and fixes the mandible and the hyoid bone, preventing the posterior rotation of these structures when the patient is in the decubitus position and thus preventing the blockage of the airways [2].

Orofacial myofunctional therapy: Orofacial myofunctional therapy (OMT) is recommended both in children and adults with mild and moderate OSA in order to improve the function of the upper airway musculature. OMT encompasses exercises that focus on various oropharyngeal structures that are involved in the upper airway collapse, such as the tongue, palate, lateral pharyngeal walls, and epiglottis, in order to establish proper respiration, speech, mastication, and deglutition. Different studies suggest that OMT has been proposed with success in reducing OSA severity, associated symptoms, and primary snoring in adults, and it is also an efficacious treatment tool for children with residual apnea [30].

#### 3.7.3. Behavioral Therapy

Informing patients correctly and engaging them in lasting lifestyle changes represent important first steps both in OSA treatment and in preventing a more severe form of the condition.

Behavioral therapy is recommended for asymptomatic patients or for those who have no apparent risk to driving safely [31]. It encompasses different strategies that help to improve the OSA-associated symptomatology, such as a regular exercise program, reducing or avoiding alcohol consumption [31], reducing or eliminating smoking [32], long-term weight reduction, and positional therapy (avoiding sleeping on one’s back) [5,31].

#### 3.7.4. Surgical Treatment

Maxillomandibular advancement or telegnathic (>10 mm) jaw advancement surgery may be indicated for patients with associated sagittal skeletal discrepancy who are unable to tolerate CPAP therapy. Surgically assisted rapid maxillary expansion (SARME) is indicated for OSA patients with maxillary transverse deficiency in order to normalize the width of the maxilla. Also, for selected patients, genioglossus advancement and hyoid suspension may be considered. Other soft tissue surgeries that may be recommended for patients with OSA include those involving the tongue, frenulum, tonsils, and adenoids [5].

#### 3.7.5. Other Treatment Options

For patients with nasal congestion or allergic rhinitis, the use of nasal steroids and other oral medication is recommended. For some patients, nasal surgery therapy is an effective way to reduce intranasal resistance and improve compliance to CPAP therapy [5].

### 3.8. Obstructive Sleep Apnea. A Patient-Centered Perspective

The interdisciplinary management of an OSA patient provides an individualized diagnosis and treatment of the condition based on the particularities of each clinical case. The relevant scientific literature has been growing considerably, allowing for the establishment of specific clinical guidelines.

A patient who is suspected of OSA must be referred to a sleep specialist, and once the OSA diagnosis is confirmed, the sleep specialist is the one who establishes the treatment plan and its stages.

The American Academy of Sleep Medicine has published a Clinical Practice Guideline for clinicians using PAP (positive airway pressure) therapy [33]. It is strongly recommended to begin PAP therapy only after the OSA diagnosis has been established based on an objective sleep apnea test. Also, proper follow-up and monitoring of the objective efficacy and use of the device should be carried out after initiating PAP therapy and during OSA treatment in order to ensure adequate adherence to the therapy [33].

Regarding the use of PAP, the American Academy of Sleep Medicine has established the following guidelines:

(1) The use of PAP is recommended, compared to no treatment, to treat OSA adult patients with excessive sleepiness (STRONG), impaired sleep-related quality of life (CONDITIONAL), and associated with comorbid hypertension (CONDITIONAL) [33].
(2) The PAP therapy should be initiated using APAP (automatic positive airway pressure) at home or in-office PAP titration in adult patients with OSA and no significant associated comorbidities (STRONG) [33].
(3) The use APAP or CPAP is recommended for the ongoing treatment of OSA adult patients (STRONG) [33].
(4) In the routine treatment of OSA adult patients, clinicians are encouraged to use CPAP or APAP over BPAP (bilevel positive airway pressure) therapy (CONDITIONAL) [33].
(5) All of the necessary instructions must be given to the OSA patient when initiating PAP therapy (STRONG) [33].
(6) During the first phase of PAP therapy in OSA adult patients, all behavioral interventions and troubleshooting must be provided (CONDITIONAL) [33].
(7) Clinicians should use telemonitoring-guided interventions during the first phase of PAP therapy in OSA adult patients (CONDITIONAL) [33].

Regarding the use of oral appliances (mandibular advancement devices), the American Academy of Sleep Medicine and the American Academy of Dental Sleep Medicine established the following clinical guidelines (2015):

(1) The use of the oral appliances is recommended, compared to no treatment, in adult patients who request treatment for primary snoring, without OSA (STANDARD) [34].
(2) When a sleep specialist prescribes an oral appliance for an OSA adult patient, it is suggested that a dental specialist use a custom, titratable device and not a non-custom oral appliance (GUIDELINE) [34].
(3) Sleep specialists should prescribe oral devices, rather than no treatment, for OSA adult patients who do not adhere to CPAP therapy or prefer an alternate treatment option (STANDARD) [34].
(4) Dental specialists should provide a follow-up of the oral appliance therapy in order to observe possible dental side effects or occlusal changes and decrease their incidence (GUIDELINE) [34].
(5) Sleep specialists should perform a follow-up sleep test in order to analyze the treatment efficacy for OSA patients with an oral appliance (GUIDELINE) [34].
(6) OSA adult patients who are undergoing oral appliance therapy must be instructed to return for periodic in-office visits with both a dental and sleep specialist (GUIDELINE) [34].

## 4. Conclusions

Obstructive sleep apnea syndrome is a prevalent disorder that affects an increasing number of both adults and children worldwide, with a great impact on the patient’s life quality, which can often be incorrectly diagnosed or can even remain undiagnosed. Adequate management is provided by an interdisciplinary medical care team which includes general practitioners and orthodontists, among other specialties. In the coming years, obstructive sleep apnea, both in adults and children, will continue to burden our society. Further research is extremely important in order to improve the diagnosis of and treatment methodologies for OSA, with the aim of serving the best interests of patients with obstructive sleep apnea syndrome.

At the moment, the subject of obstructive sleep apnea syndrome is not included in the curricula of most dental schools or in orthodontic and dentofacial orthopedic residencies. Given the prevalence and importance of this condition, it is strongly recommended to consider developing a standardized curriculum and introducing it to dental universities and residency programs worldwide.

To improve the diagnosis and treatment of OSA, it is mandatory that patients be approached by a multidisciplinary medical care team, which provides strong collaboration between the general specialists, orthodontists, and other specialties.

## 5. Executive Summary

Obstructive sleep apnea syndrome represents an increasingly prevalent medical condition that can have important consequences on an individual’s life if it is left undiagnosed and thus untreated. OSA is linked to serious medical comorbidities such as myocardial infarction, heart failure, hypertension, and global cardiovascular morbidity. Commonly, it affects male patients of 45–65 years of age, but recently, the number of children affected by this disorder has increased. Snoring and daytime sleepiness are among the most common symptoms, but many patients with OSA are asymptomatic. The diagnosis of OSA should be carried out with the use of PSG, while 3D imaging examinations such as CBCT and MRI only have to be used for monitoring or treatment considerations.

It is very important for orthodontists and general dentists to be familiar with the signs and symptoms of this disorder, so if they have a clinical suspicion that a patient may have OSA, they can refer them to a sleep medicine physician for further evaluation and a definitive diagnosis. It is highly recommended that the management of an OSA patient be carried out by a multidisciplinary team that includes specialists from different health areas: pneumology, cardiology, neurology, ENT, psychiatry, orthodontics and dentofacial orthopedics, and oral maxillofacial surgery. The decision on the treatment sequences will be made only by the sleep medicine specialist. This interdisciplinary approach maximizes the treatment outcomes of OSA patients. Asymptomatic OSA patients or those who have no apparent risk to driving safely can be treated with behavioral interventions such as a regular physical exercise program and weight loss, while CPAP therapy is indicated for patients with more severe symptomatology and associated comorbidities. Mandibular advancement devices and myofunctional therapy represent treatment options for patients with mild to moderate OSA. Patients with OSA and associated craniofacial abnormalities can undergo oral maxillofacial surgery.

## Data Availability

Not applicable. No new data were created in this study.
